# Impact of Implant Site and Bone Quality on the Accuracy of Robot‐Assisted Implant Placement: A Retrospective Study

**DOI:** 10.1155/ijod/3947015

**Published:** 2026-02-17

**Authors:** Jianfei Liang, Jiayin Li, Ningbo Zhao, Lifan Liao, Wei Liang, Yibing Liu, Longlong He, Qin Zhou

**Affiliations:** ^1^ Key Laboratory of Shaanxi Province for Craniofacial Precision Medicine Research, College of Stomatology, Xi’an Jiaotong University, Xi’an, China, xjtu.edu.cn; ^2^ Clinical Research Center of Shaanxi Province for Dental and Maxillofacial Disease, College of Stomatology, Xi’an Jiaotong University, Xi’an, China, xjtu.edu.cn; ^3^ Department of Implant Dentistry, College of Stomatology, Xi’an Jiaotong University, Xi’an, China, xjtu.edu.cn

**Keywords:** dental implants, dimensional measurement accuracy, robotics

## Abstract

**Background:**

Robot‐assisted surgery is widely used in implant dentistry; however, the specific effects of implant site and bone quality on its accuracy require further elucidation.

**Objective:**

This study aimed to evaluate the impact of implant site and bone quality on the accuracy of a single robotic‐assisted implant placement method.

**Methods:**

This retrospective analysis included 83 patients who received 173 implants via a robot‐assisted system. Implants were categorized by site into upper‐anterior (UA), upper‐posterior (UP), lower‐anterior (LA), and lower‐posterior (LP) regions and by bone quality into Type I–II and Type III–IV. Postoperative accuracy was assessed by comparing the planned versus actual implant positions, measuring lateral coronal deviation (LCD), vertical coronal deviation (VCD), and global coronal deviation (GCD); lateral apical deviation (LAD), vertical apical deviation (VAD), and global apical deviation (GAD); and angular deviation (AD).

**Results:**

When analyzed by implant site, the UA region demonstrated significantly larger vertical deviations (VCD and VAD) compared to the LP region (VCD: 0.99 ± 0.46 mm vs. 0.25 ± 0.53 mm, *p*  < 0.05; VAD: 0.95 ± 0.46 mm vs. 0.24 ± 0.53 mm, *p*  < 0.05). AD did not differ significantly among the various implant sites. Regarding bone quality, however, implants in Type III–IV bone exhibited significantly greater vertical deviations than those in Type I–II bone (VCD and VAD: 0.67 ± 0.56 mm vs. 0.27 ± 0.49 mm, *p*  < 0.05). Moreover, a trend toward a larger AD was also noted in Type III–IV bone (1.53 ± 1.09° vs. 1.18 ± 0.80°).

**Conclusions:**

Both implant site and bone quality were found to influence the accuracy of the single robotic‐assisted system.

## 1. Introduction

Dental implants have become the preferred treatment modality for the replacement of missing teeth. The ideal three‐dimensional (3D) position of the implant mainly depends on the appropriate planning and accurate implantation surgery, which is the key to the extension of the service life of the implant [1]. Surgical planning is usually needed to evaluate the alveolar bone quality and the location of the alveolar nerve or vessel accurately through 3D imaging techniques such as cone‐beam computed tomography (CBCT), computer‐assisted scans, and implant design software [2, 3]. Preoperative planning is essential for defining a safe implant position to protect neurovascular structures. However, during surgery, the clinician relies on mental imagery of the bone architecture, making the accurate translation of the planned position to the operative site difficult [4]. Consequently, this results in an increased risk of surgical failure and reduces the service life of the implant [1]. Therefore, improving implantation accuracy is of great significance to successful implant surgery, and it is also an important direction of oral digital development at present [5].

Computer‐assisted implant surgery (CAIS) provides a personalized, predictable, and efficient surgical approach for the precise implantation of implants [6]. CAIS includes static guides, dynamic navigation, and robotics [6, 7]. Notably, static guides can improve the accuracy of implant surgery to a certain extent compared with free hand [8]. However, it is mainly faced with high production costs, long cycles, difficulty in intraoperative adjustment, and error accumulation from the design [9]. Dynamic navigation also requires the doctor to operate the navigation system and the operation simultaneously, which may increase the complexity and difficulty of the operation, and it is difficult to avoid hand tremors and inaccurate perception [10, 11]. In contrast, robotic assistance ensures precise drill positioning and angulation while offering greater accuracy and reproducibility than conventional approaches [12]. In addition, the requirement for surgical experience or skills is relatively lower, and the learning curve of doctors is shorter than that of dynamic navigation [13]. Therefore, robot‐assisted placement has been widely used in implant surgery [14].

Various studies have reported the accuracy of robot‐assisted implant placement in different types of surgery, such as immediate implantation and single‐tooth or edentulous implantation [15–17]. A study by Scotty et al. involving 38 implants reported a mean angular deviation (AD) of 2.56 ± 1.48° and a deviation from the plan of 1.04 ± 0.70 mm for the surgical robot (Yomi, Neocis Inc., Miami, FL, USA) [15]. Similarly, Yang et al. [17] found that robotic assistance with the robotic dental surgery system (Remebot, Beijing Baihui Weikang Technology Co., Ltd., China) achieved an AD of 1.27° ± 0.59° and a global coronal deviation (GCD) of 0.67 ± 0.37 mm in a cohort of 10 patients with 59 implants. However, it is well‐known that bone quality is essential in preoperative evaluation, and loose or hard bone will affect the implant osseointegration [18, 19]. Likewise, the space or bone quality of different implant sites isdifferent, which may lead to the deviation of implantation. For example, in the narrow space of the mouth, the space of the posterior tooth is smaller than that of the anterior tooth [20]. Compared with the mandibular, the maxillary bone quality was relatively poor [19]. Type D4, according to the Misch bone density classification, has poor density with trabeculae strength 10 times weaker than Type D1 [21]. Currently, the effects of different implant sites and bone quality on the accuracy of robot‐assisted implantation remain unclear. Therefore, it is necessary to compare the accuracy in conditions of different implant sites and bone quality, which has reference significance for surgical evaluation before applying robot‐assisted implantation.

## 2. Material and Methods

### 2.1. Study Design and Patient Selection

This single‐center retrospective study was approved by the Ethics Committee of Stomatology at Xi’an Jiaotong University (Approval No. 2023‐037‐003) and conducted in accordance with the Declaration of Helsinki, US Federal Policy for the Protection of Human Subjects, and the European Medicines Agency Guidelines for Good Clinical Practice. The study analyzed data from consecutive patients who underwent robot‐assisted implant placement by a single surgeon (with over 10 years of clinical experience) at the Department of Stomatology, Xi’an Jiaotong University Affiliated Hospital, between July 10, 2023, and August 10, 2024.

Patient records were retrieved from the database of the Department of Implant Dentistry. A total of 83 patients (173 implants) were initially enrolled, irrespective of gender. All participants met the following criteria.

### 2.2. Inclusion Criteria

a. Adequate bone volume and density at the implant site, obviating the need for bone grafting;
b. Agreement to undergo robot‐assisted implant surgery;
c. Good general health without significant systemic diseases;
d. Adequate mouth opening and absence of tooth mobility in the remaining dentition.

### 2.3. Exclusion Criteria

a. Incomplete preoperative or postoperative CT data;
b. Poor alveolar bone conditions requiring bone grafting during surgery;
c. History of bone grafting at the intended implant site.

### 2.4. Workflow and Procedure of Robot‐Assisted Implantation

One commercial robotic system was employed in this study (Remebot, Beijing Baihui Weikang Technology Co., Ltd., Beijing, China). The operating principle of the Remebot Robot is shown in Supporting Information: Figures. The workflow of robot‐assisted computer‐aided implant surgery (r‐CAIS) in this study comprised three consecutive phases: preoperative preparation, intraoperative surgical execution, and postoperative verification. (a) Preoperative planning: A custom positioning marker (Remebot, Beijing) was fixed to the patient’s dental arch. CBCT (Orthophos XG, Dentsply Sirona, Germany) scan was then performed with the marker in place. The CBCT scanning parameters were 110 kV/5 mA with 0.3 mm voxel size and 12 s scanning time. The digital imaging and communications in medicine (DICOM) data were imported into the robotic planning software, where the implant position, drilling path, and sequence were virtually designed based on prosthetic guidelines and bone anatomy. (b) Intraoperative registration and surgery: Spatial registration was achieved using an optical tracking system. The preoperative positioning markers were secured to the patient’s dental arch using a self‐cured acrylic resin (Protemp™, 3M ESPE, Neuss, Germany). The reflective spheres on the fixed marker served as fiducial points, allowing the system to dynamically track the patient’s position and align the preoperative plan with the physical surgical field in real‐time. Following successful registration, the robotic arm autonomously executed the planned osteotomy, with the surgeon initiating and controlling the drilling. The implant was then placed according to the plan site using robotic assistance, without manual insertion. Straumann® BLT implants (Institut Straumann AG, Basel, Switzerland) and the ASTRA Tech Implant System (Dentsply Sirona Implants, Mölndal, Sweden) implants were mainly used. (c) Postoperative assessment: A postoperative CBCT scan was acquired. By superimposing this scan onto the preoperative plan, the deviations between the planned and actual implant position were measured to evaluate procedural accuracy. The procedures following surgery were conducted similarly to our previously published work [22]. The patients were monitored for a period of 6 months postoperatively, and no complications were observed during this time.

### 2.5. Classification for Bone Types

The classification is based on the classification by Lekholm and Zarb (1985). Class I: Almost entirely composed of compact bone. Class II: Thick dense bone surrounds the high‐density spongy bone. Class III: A thin layer of dense bone surrounds the low‐density cancellous bone. Class IV: A thin dense bone surrounds the low‐density spongy bone. The bone type classification was assessed by two doctors, each with over 3 years of dedicated clinical experience and both co‐authors of this study; the assessments were performed while blinded to other clinical information.

### 2.6. Accuracy Assessment

The postoperative CBCT of DICOM files was introduced into the preoperative planning of the robot software (Remebot, Beijing) and fitted with the preoperative CBCT image. In the postoperative verification section of the software, the software automatically extracted the postoperative implant contour. Subsequently, it was fitted with the planned virtual implant to automatically calculate the deviation between the planned and placed implants. The analyzed coronal or apical deviation includes the global, lateral, and vertical deviations, which were calculated respectively by the long axis of the planned implant and placed implant, and the AD was also calculated. Regarding the central axes of the planned versus placed implants, the accuracy data indicated deviations in mm, including global, vertical, coronal, lateral, and apical deviations.

### 2.7. Statistical Analysis

Statistical analyses were performed using GraphPad PRISM software version 8.0.2 (GraphPad Software, Inc., San Diego, US). The quantitative data were shown as mean ± standard deviation (SD). Tukey’s multiple comparisons test was used to compare the data between the two groups by SPSS Statistics version 26.0 (SPSS, Armonk, NY, USA). Two‐way analysis of variance (ANOVA) (two‐factor analysis) was also conducted. ANOVA or the Kruskal–Wallis test was used (Supporting Information 2). Given the retrospective nature of this study, a pre hoc sample size calculation was not feasible. However, a post hoc power analysis was conducted on the primary outcome of vertical coronal deviation (VCD) between bone quality groups, which demonstrated a power of >80% to detect the observed difference at the = 0.05, indicating that the sample size was adequate. The significance level for all statistical tests was set at *p*  < 0.05.

## 3. Results

### 3.1. Patient Information and Experimental Design

The demographic and surgical data of the subjects are presented in Supporting information: Table S1. This study retrospectively and randomly analyzed 83 cases of robot‐assisted implant placement, comprising 49 males and 34 females, aged 20–77 years. All implant osteotomy preparations and placements were performed with robotic assistance. No intraoperative adverse events were recorded, and no postoperative complications such as infection or implant failure were observed during the 6‐month follow‐up.

Implant sites were classified into four regions: upper‐anterior (UA: teeth 13–23), upper‐posterior (UP: teeth 14–17, 24–27), lower‐anterior (LA: teeth 33–43), and lower‐posterior (LP: teeth 34–37, 44–47) (Figure 1A). Accuracy was evaluated by comparing planned versus actual implant positions, measuring linear deviations (coronal, apical, and global), vertical deviations (coronal, apical), and AD (Figure 1B).

Figure 1Schematic diagram of implant sites and analytical deviation. (A) Implant sites were divided into four regions: upper‐anterior (UA) was indicated by green implants; dark blue implants indicated upper‐posterior (UP); lower‐anterior (LA) was indicated by cyan implants; and lower‐posterior (LP) was indicated by red implants. (B) Schematic diagram of the accuracy analysis of the planned and placed implants. The pink implant represents the placed implant; gray implant represents the planned implant. The analyzed parameters include LCD, lateral coronal deviation (a), VCD, vertical coronal deviation (b), GCD, global coronal deviation (c), LAD, lateral apical deviation (d), VAD, vertical apical deviation (e), GAD, global apical deviation (f), and AD, angular deviation (g).

**Figure:**
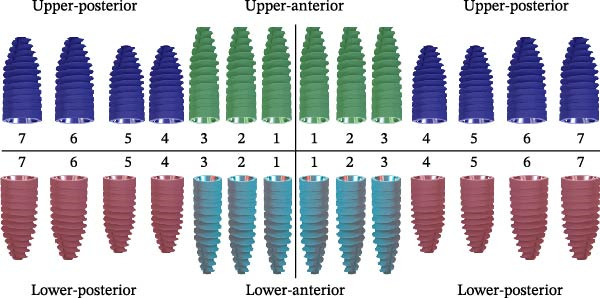
(A)

**Figure:**
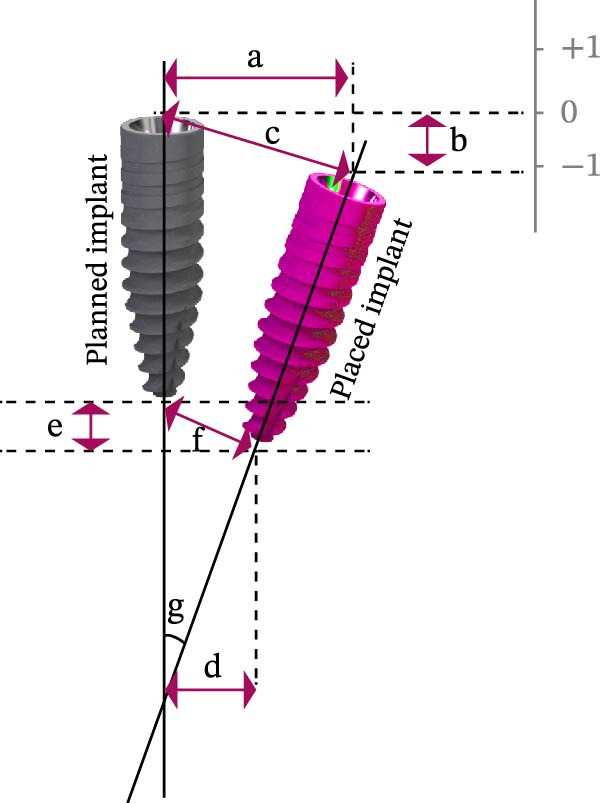
(B)

### 3.2. Distance Deviation in Different Implant Sites

The analysis of lateral coronal deviation (LCD) revealed no significant differences among the four implant regions (UA: 0.36 ± 0.20 mm; UP: 0.31 ± 0.15 mm; LA: 0.41 ± 0.17 mm; and LP: 0.37 ± 0.19 mm). In contrast, VCD in the UA region was significantly greater than in the LP region (0.99 ± 0.46 mm vs. 0.25 ± 0.53 mm), while no difference was found between UP and LP (0.61 ± 0.45 mm vs. 0.25 ± 0.53 mm). For GCD, the UA region showed significantly greater deviation than both the LP (1.05 ± 0.46 mm vs. 0.64 ± 0.33 mm) and UP (1.05 ± 0.46 mm vs. 0.77 ± 0.30 mm) regions (Figure 2A–C; Table 1).

Figure 2Analysis of implant accuracy in different implant sites. A–F Analysis of lateral coronal deviation (A), vertical coronal deviation (B), global coronal deviation (C), lateral apical deviation (D), vertical apical deviation (E), and global apical deviation (F) at implant sites of upper‐anterior, upper‐posterior, lower‐anterior, and lower‐posterior.

**Figure:**
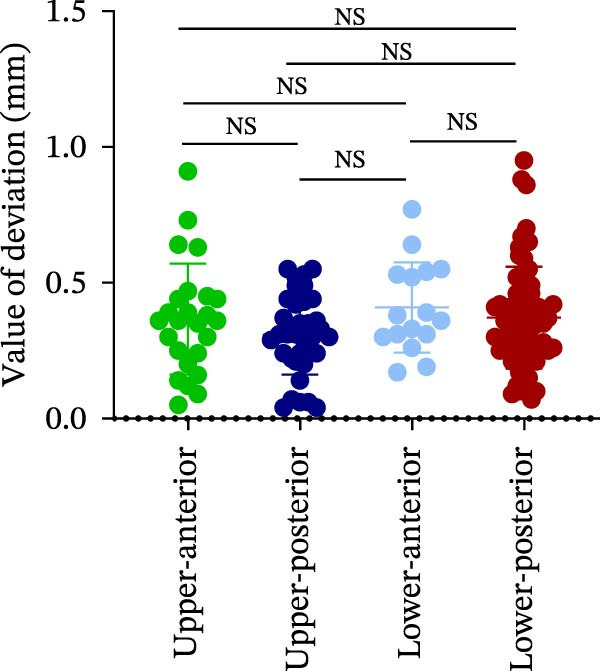
(A)

**Figure:**
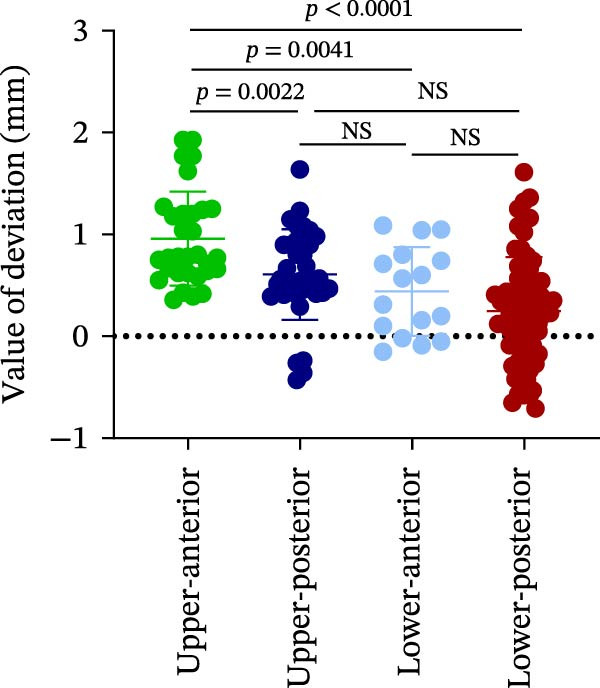
(B)

**Figure:**
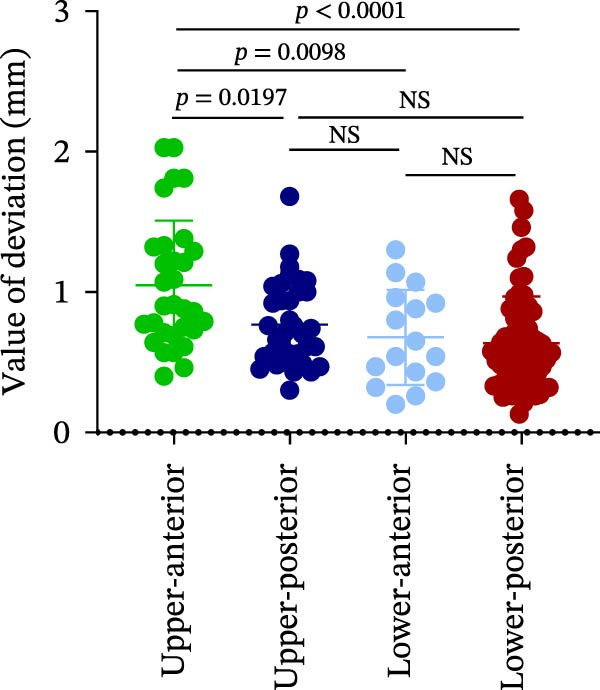
(C)

**Figure:**
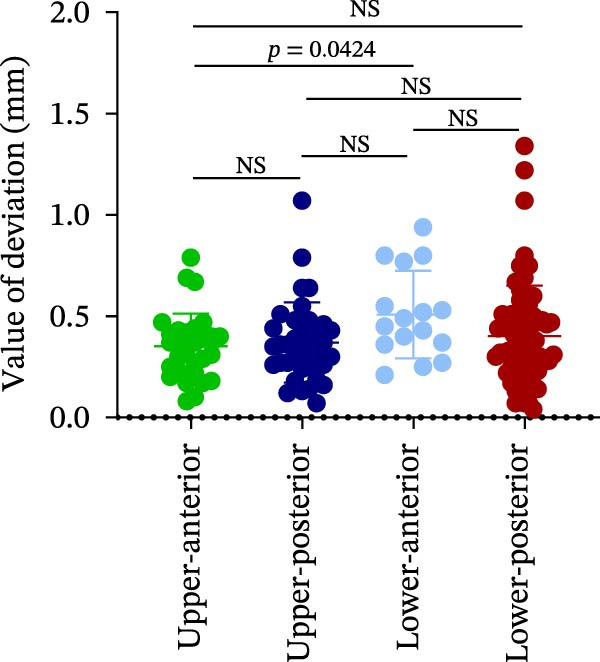
(D)

**Figure:**
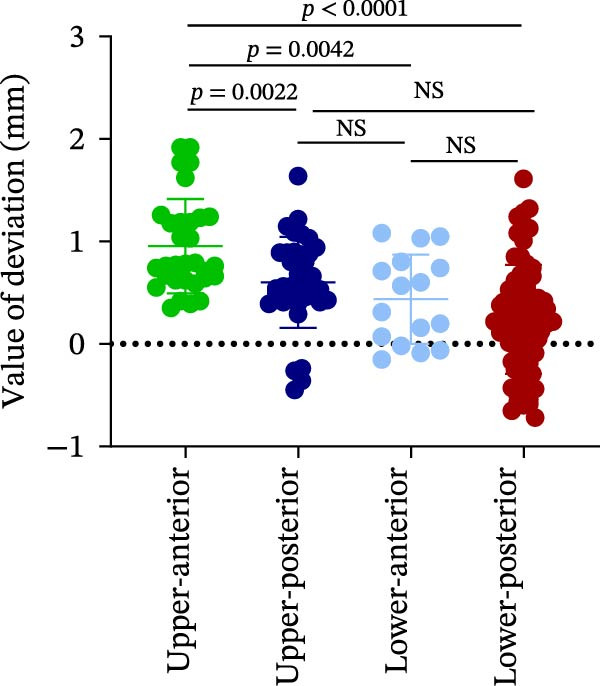
(E)

**Figure:**
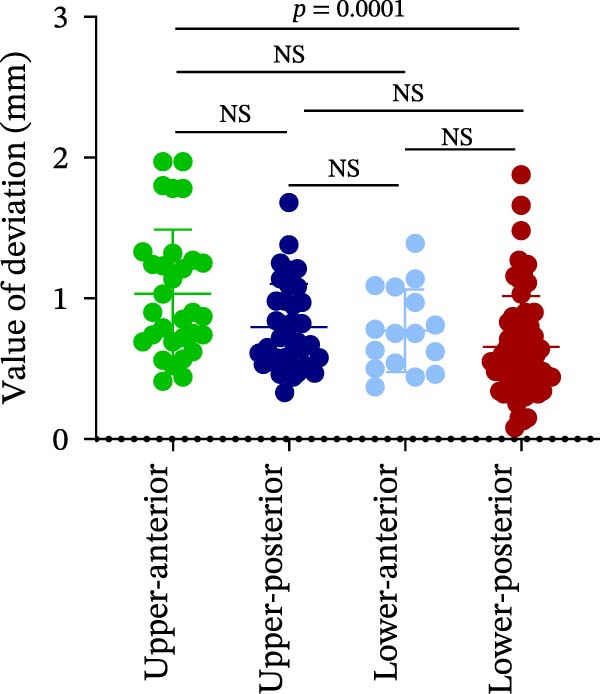
(F)

**Table 1:** Deviation distance of robot‐assisted implantation in different implant site and bone quality.

	Implant sites				Bone quality	
(Mm)	UA	UP	LA	LP	I–II	III–IV
LCD	0.36 ± 0.20	0.31 ± 0.15	0.41 ± 0.17	0.37 ± 0.19	0.34 ± 0.18	0.37 ± 0.19
VCD	0.99 ± 0.46	0.61 ± 0.45	0.44 ± 0.44	0.25 ± 0.53	0.27 ± 0.49	0.67 ± 0.56
GCD	1.05 ± 0.46	0.77 ± 0.30	0.68 ± 0.34	0.64 ± 0.33	0.61 ± 0.30	0.87 ± 0.41
LAD	0.35 ± 0.16	0.37 ± 0.20	0.51 ± 0.22	0.40 ± 0.25	0.39 ± 0.22	0.41 ± 0.22
VAD	0.95 ± 0.46	0.60 ± 0.44	0.44 ± 0.44	0.24 ± 0.53	0.27 ± 0.49	0.67 ± 0.56
GAD	1.04 ± 0.46	0.80 ± 0.31	0.77 ± 0.29	0.66 ± 0.36	0.65 ± 0.29	0.89 ± 0.42

Abbreviations: GAD, global apical deviation; GCD, global coronal deviation; LA, lower‐anterior; LAD, lateral apical deviation; LCD, lateral coronal deviation; LP, lower‐posterior; UA, upper‐anterior; UP, upper‐posterior; VAD, vertical apical deviation; VCD, vertical coronal deviation.

Then, the apical deviation in different implant sites was also compared. We found that there were no differences in lateral coronal deviation (LAD) in different implant sites, similar to LCD (Figure 2D). However, the vertical apical deviation (VAD) in UA was greater than that in LP (0.95 ± 0.46 mm vs. 0.24 ± 0.53 mm) (Figure 2E; Table 1). Furthermore, there was no difference between the VAD in UP and that in LP (0.60 ± 0.44 mm vs. 0.24 ± 0.53 mm) (Figure 2E; Table 1). By analyzing the global apical deviation (GAD), the deviation of UA was greater than that in LP (1.04 ± 0.46 mm vs. 0.66 ± 0.36 mm) (Figure 2F; Table 1). In summary, vertical deviations (both coronal and apical) were more susceptible to variation depending on implant site, with UA region consistently exhibiting greater deviation than the LP region in robot‐assisted implant placement.

### 3.3. Distance Deviation in Different Bone Quality

To assess the impact of bone quality on the accuracy of robot‐assisted implant placement, bone density was categorized into Class I–II and Class III–IV based on CBCT findings, and the corresponding positional deviations were analyzed. Class I–II bone was defined as dense or porous cancellous bone encased in a thick cortical layer, whereas Class III–IV bone was defined as dense or porous cancellous bone surrounded by a thin cortical layer (Figure 3A; Table 1). We found that there were no differences in LCD (0.34 ± 0.18 mm vs. 0.37 ± 0.19 mm) and LAD (0.39 ± 0.22 mm vs. 0.41 ± 0.22 mm) in bone density with Class I–II and III–IV (Figure 3B, E; Table 1). However, the VCD (III–IV: 0.67 ± 0.56 mm vs. I–II: 0.27 ± 0.49 mm) and GCD (III–IV: 0.87 ± 0.41 mm vs. I–II: 0.61 ± 0.30 mm) in Type III–IV were greater than those in bone quality with Type I–II (Figure 3C, D; Table 1). Moreover, the VAD (III–IV: 0.67 ± 0.56 mm vs. I–II: 0.27 ± 0.49 mm) and GAD (III–IV: 0.89 ± 0.42 mm vs. I–II: 0.65 ± 0.29 mm) in Type III–IV bone were greater than those in bone quality with Type I–II (Figure 3F, G; Table 1). These results suggest that Class III–IV bone quality is more prone to induce vertical deviation errors compared to Class I–II bone during robot‐assisted implant placement.

Figure 3Analysis of accuracy in different bone quality. (A) Bone quality was divided into four grades: I, II, III, and IV. Representative images showed the bone quality with Class I–II (a) and Class III–IV (b). (B–G) Analysis of lateral coronal deviation (B), vertical coronal deviation (C), global coronal deviation (D), lateral apical deviation (E), vertical apical deviation (F), and global apical deviation (G) at implant site with bone quality of I–II and III–IV.

**Figure:**
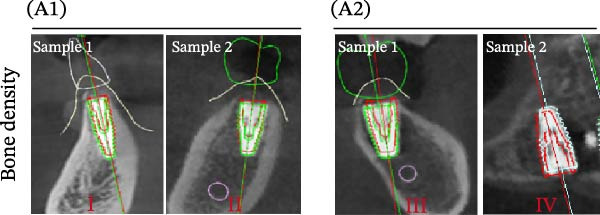
(A)

**Figure:**
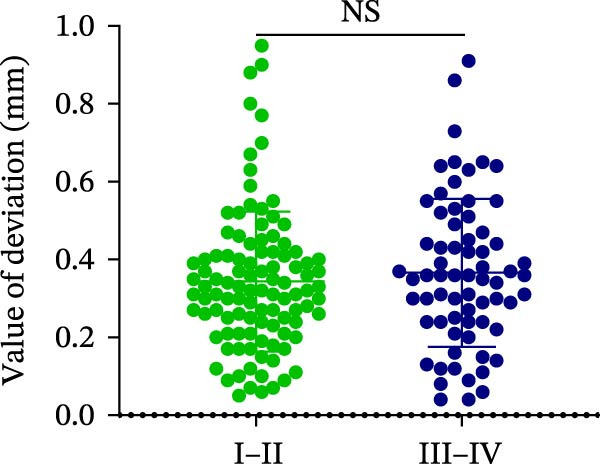
(B)

**Figure:**
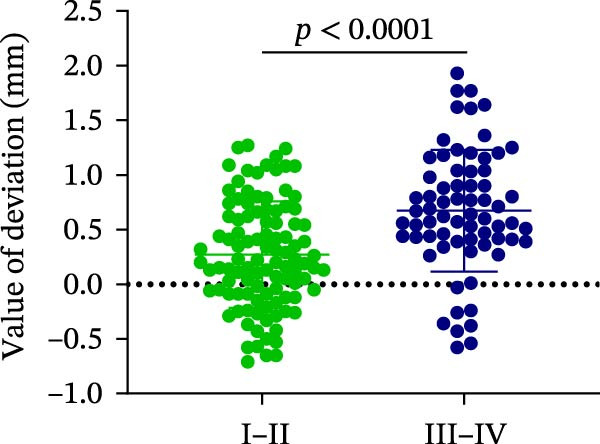
(C)

**Figure:**
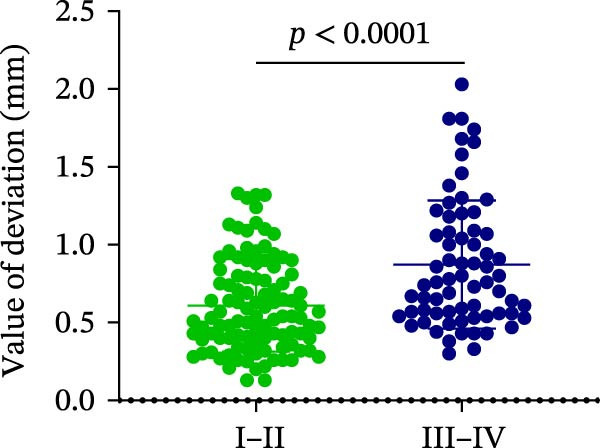
(D)

**Figure:**
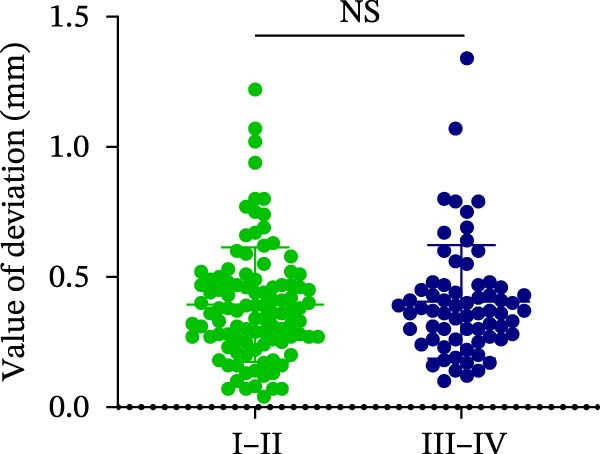
(E)

**Figure:**
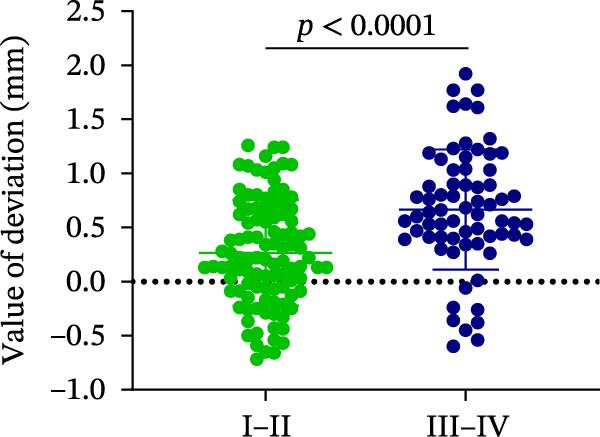
(F)

**Figure:**
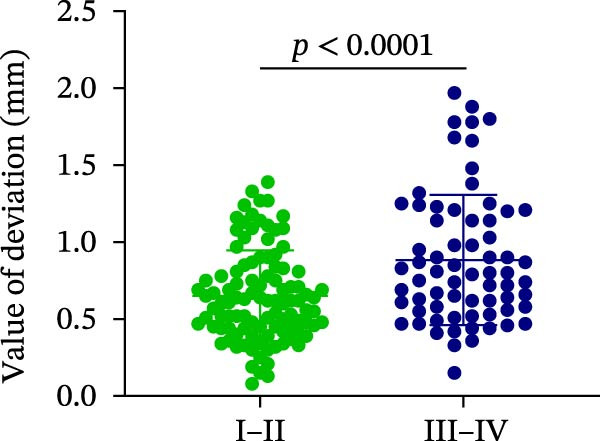
(G)

### 3.4. AD in Different Implant Sites and Bone Quality

Finally, AD was analyzed in relation to different implant sites and bone quality in robot‐assisted implantation. The results showed no significant differences in AD among the UA, UP, LA, and LP sites (1.15 ± 0.47° vs. 1.42 ± 1.20° vs. 1.06 ± 0.72° vs. 1.40 ± 1.18°) (Figure 4A; Table 2). However, AD was significantly greater in Type III–IV bone than in Type I–II bone (1.53 ± 1.09° vs. 1.18 ± 0.80°) (Figure 4B; Table 2). This suggests that poorer bone quality (Type III–IV) can increase AD in robot‐assisted implant placement.

Figure 4Angular deviation in different implant sites and different bone quality. (A) Angular deviation of implant sites at upper‐anterior, upper‐posterior, lower‐anterior, and lower‐posterior. (B) Angular deviation of implanted sites with Class I–II and Class III–IV bone quality.

**Figure:**
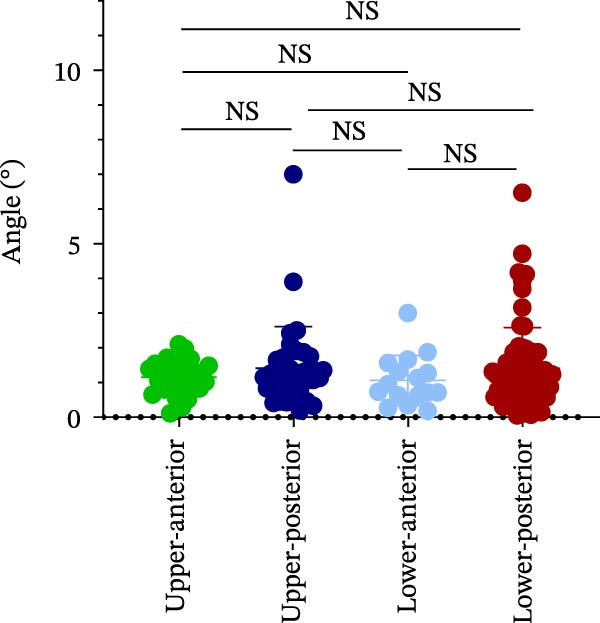
(A)

**Figure:**
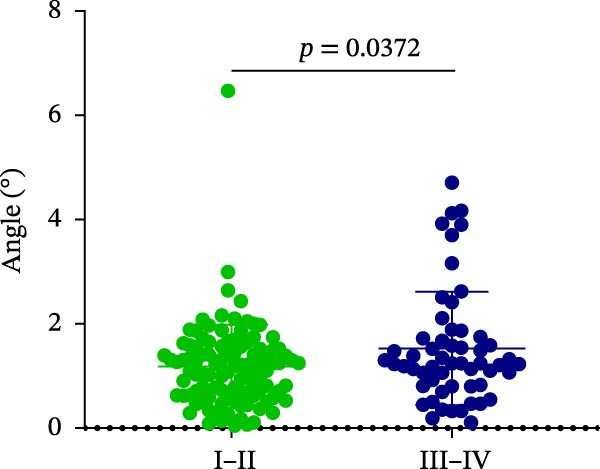
(B)

**Table 2:** Angular deviation of robot‐assisted implantation in different implant site and bone quality.

	Implant location				Bone quality	
(°)	UA	UP	LA	LP	I–II	III–IV
Angle	1.15 ± 0.47	1.42 ± 1.20	1.06 ± 0.72	1.40 ± 1.18	1.18 ± 0.80	1.53 ± 1.09

Abbreviations: LA, lower‐anterior; LP, lower‐posterior; UA, upper‐anterior; UP, upper‐posterior.

## 4. Discussion

Our data indicate a significant association between poorer bone quality and increased vertical and ADs. This finding underscores that regional anatomical challenges—including bone density, arch curvature, and surgical access—persist despite robotic assistance and require careful consideration during planning and execution. The vertical deviation in the UA significantly exceeded that of the LP, which contributes to overall global deviation. The observed differences in accuracy between the UA and LP are likely attributable to a combination of marker stabilization or variations in bone thickness and morphology. Nevertheless, the maximum observed deviation remained below 2 mm, demonstrating superior precision to the largest deviations reported in prior CAIS studies [23]. However, the different implant sites did not affect the lateral distance deviation. Moreover, the average deviation was less than 1 mm. Different implant sites did not affect the AD, all of which were less than 2°. The vertical deviation of bone quality with Type III–IV was significantly greater than that of Type I–II, and the maximum deviation was less than 2 mm. Nevertheless, the bone density did not affect the lateral distance deviation; the average deviation was less than 0.5 mm. Bone quality slightly affected the AD, which was less than 2°. These deviations meet the acceptable deviation in clinical implantation, which has advantages over navigation and guides [24, 25]. Furthermore, comparing the sites and bone quality allows us to consider the possible deviation when using robot‐assisted implantation, which is helpful for clinical guidance.

Bone quality, which varies across different implant sites, is a key determinant of implant primary stability [26, 27]. For example, Type III bone quality was more frequent in the posterior (73.33%) and anterior (73.33%) sites in the maxilla [26]. There are several possible reasons for the deviation of vertical distance under different bone densities: (a) After implantation, the doctor needs to adjust the implant depth due to unexpected deviations caused by CBCT [28, 29]; (b) Controlling the vertical depth when the planting robot arm goes into the III–IV bone is difficult due to the soft bone tissue without resistance [30]; (c) Deviation during removal of the implant carrier due to inadequate retention. Furthermore, the angle deviation is relatively large in the case of poor bone density, but the overall difference is not large. Ramadhan et al. reported that low bone density in the implant placement site might be a risk factor influencing the accuracy of implant placement with computer‐guided surgery [31]. The main cause is likely dislocation resulting in implant displacement [32, 33]. Notably, Abduo and Lau’s [34] findings indicate that regardless of guidance protocol (fully or pilot‐guided), static computer‐assisted implant placement achieves less precise outcomes in posterior sites than in anterior regions.

The accuracy of robot‐assisted implant placement observed in our study is consistent with the range reported in the literature, yet a critical analysis reveals important nuances related to patient selection and clinical protocol. When compared with the seminal work by Bolding [15] et al. (deviation from the plan of 1.04 ± 0.70 mm, AD of 2.56 ± 1.48°), our results fall within a comparable range. The slightly higher deviations reported in their cohort might be attributable to differences in case complexity and the different robotic systems of Yomi. In contrast, the superior accuracy reported by Yang et al. [17] (AD of 1.11°) and Zhao et al. [22] (AD of 1.17°) can be contextualized by their specific clinical parameters. Yang et al.’s study was exclusively based on single‐tooth implant placement in the anterior region, which typically offers better access, bone volume, and surgeon visibility. Similarly, Zhao et al. [22] focused on immediate implant placement, which may involve different biomechanical and surgical considerations. Therefore, the lower deviations in these studies likely reflect the less challenging clinical scenarios rather than an inherent superiority of the robotic systems. Our results, derived from a potentially more varied cohort, provide a complementary perspective on the technology’s performance under broader clinical conditions.

This study has a theoretical guiding role for the clinical use of robot‐assisted implantation surgery. First, we found that the angle deviation with robot assistance is less than 2°, which is far superior to the accuracy of surgical guides and dynamic navigation [35–38]. As such, we need to design good sites, and it is unnecessary to be concerned about the angle deviation that the robot brings. In the process of general implantation, it is unnecessary to be concerned about the lateral distance deviation of robot implantation under different implant sites or bone quality since all deviations are about 1 mm. We should also pay more attention to the vertical distance deviation; the vertical distance deviation of the UA exhibited a significantly larger deviation, and the deviation of the Type III–IV bone may also be even larger. When encountering the UA or Type III–IV bones, we should be more cautious and reserve enough vertical safety distance greater than 3 mm to avoid impairing important anatomical structures such as the maxillary sinus floor, blood vessels, and nerves. In conclusion, although robot implantation has recognized biases, they are far more accurate than guides and navigation. The results of this study supplement the theoretical guidance for fine guidance on the clinical use of machine‐assisted implantation surgery.

This study has several limitations. First, it was a single‐center investigation with a relatively limited sample size. Second, 173 implant sites were randomly selected, and the number of cases needs to increase further. Finally, this study focused on evaluating the performance of a single system (Remebot®) without a direct, controlled comparison to other robotic platforms. The deviation distance summarized in this study is based on the operational proficiency of the surgeons in this research team, and there may be some precision deviation in different operations or resorbed ridge with a steep slope [39]. Therefore, our findings primarily apply to other optically navigated, surgeon‐controlled robotic systems (e.g., Yomi, Navident) and should not be directly extended to mechanically guided or fully autonomous platforms. Future comparative studies are needed to define broader benchmarks and platform‐specific traits. The surgical procedure or implant brand or implant size may also affect the accuracy. The use of a qualitative classification without interobserver calibration is also a limitation. Notably, while the emergence of autonomous robots such as Yakebot may improve these conditions [40], future studies should specifically pursue multicenter validation with larger samples, conduct comparative trials among different robotic systems, and explore the application and evaluation of autonomous platforms in complex anatomical scenarios.

Future comparative studies across different robotic classes are essential to establish universal principles and distinguish platform‐specific performance characteristics.

## Author Contributions

Jianfei Liang, Qin Zhou, and Longlong He contributed to the design, data analysis, and drafting article. Jiayin Li, Wei Liang, and Yibing Liu contribued to data analysis, drafting article, and data collection. Lifan Liao, Ningbo Zhao, and Jiayin Li contributed to the approval of article, statistics, and data collection. Funding was secured by Jianfei Liang, Qin Zhou, and Longlong He.

## Funding

The study was financially supported by the Shaanxi Province Natural Science Basic Research Program (Grant 2024JC‐YBQN‐0974) and the Xi’an Jiaotong University Basic Scientific Research Business Funds (Grant xzy012024110).

## Conflicts of Interest

The authors declare no conflicts of interest.

## Supporting Information

Additional supporting information can be found online in the Supporting Information section.

## Supporting information


**Supporting Information 1** Table S1: Participant demographic and surgical information divided into two groups by bone quality.

**Supporting Information 2** Table S2: Participant demographic and surgical information divided into four groups by implant site.

## Data Availability

The datasets used and/or analyzed in the current study are available from the corresponding author upon reasonable request.
