# Supplementary material for: Impact of Implant Site and Bone Quality on the Accuracy of Robot‐Assisted Implant Placement: A Retrospective Study
Source: Int J Dent. 2026 Feb 17;2026:3947015. doi: 10.1155/ijod/3947015 (PMC12913689; doi:10.1155/ijod/3947015)
Supplement: Supplementary file 1 — Supporting Information 1 Table S1: Participant demographic and surgical information divided into two groups by bone quality. [file IJOD-2026-3947015-s002.docx]

**Supplementary Figure.**


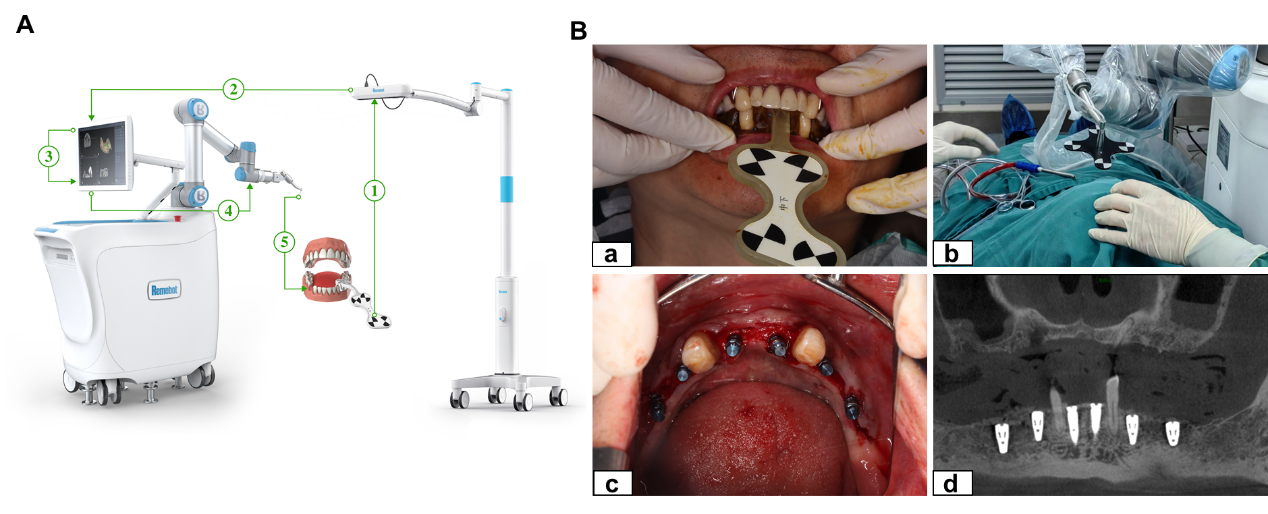


1. The operating principle of Remebot Robot. ① The optical tracking positioning instrument is used to real-time locate the patient's posture during the operation.② The patient's posture is transmitted in real time to the robot implant software system.③ The system software combines the patient's posture, CBCT, and implant planning to generate real-time control instructions.④ The system software sends the robot motion control instructions to the mechanical arm.⑤ The robot's end effector moves to the planned implant position and completes the surgical action.
2. The representative clinical images of implantation procedure: a. Wearing a marker in the mouth; b. Install the marker of the mechanical arm; c. Photo of implant after implantation; d. Radiographic image of implant.

**Supplementary Table 1. Participant demographic and surgical information** **divided into two groups by bone quality**

| Bone types Number of samples,N; Proportion(%) | Subject No. | Sex | Age  (Years old) | Implant location | Bone quality  type | Company | Implant Diameter×Length (mm) |
| --- | --- | --- | --- | --- | --- | --- | --- |
| III~IV  (N=69; 39.9%) | 1 | F | 31 | 21 | III | Straumann BLT | 3.3×10 |
|  | 2 | M | 23 | 11 | III | Straumann BLT | 3.3×10 |
|  | 2 | M | 23 | 21 | III | Straumann BLT | 3.3×10 |
|  | 3 | M | 47 | 16 | III | Straumann BLT | 4.8×10 |
|  | 4 | F | 40 | 11 | IV | Straumann BLT | 3.3×10 |
|  | 5 | F | 43 | 11 | IV | Straumann BLT | 3.3×12 |
|  | 6 | F | 65 | 11 | IV | Straumann BLT | 3.3×12 |
|  | 6 | F | 65 | 12 | IV | Straumann BLT | 3.3×10 |
|  | 6 | F | 65 | 14 | IV | Straumann BLT | 3.3×10 |
|  | 6 | F | 65 | 16 | IV | Straumann BLT | 4.8×10 |
|  | 7 | F | 53 | 17 | IV | Straumann BLT | 4.8×10 |
|  | 7 | F | 53 | 17 | IV | Straumann BLT | 4.8×8 |
|  | 7 | F | 53 | 21 | IV | Straumann BLT | 3.3×12 |
|  | 8 | F | 54 | 23 | IV | Straumann BLT | 3.3×10 |
|  | 8 | F | 54 | 24 | IV | Straumann BLT | 4.1×10 |
|  | 8 | F | 54 | 25 | IV | Straumann BLT | 4.1×10 |
|  | 9 | F | 43 | 26 | IV | Straumann BLT | 4.8×8 |
|  | 9 | F | 43 | 27 | IV | Straumann BLT | 4.8×10 |
|  | 9 | F | 43 | 27 | IV | Straumann BLT | 4.8×8 |
|  | 10 | M | 60 | 21 | III | Straumann BLT | 3.3×12 |
|  | 11 | F | 24 | 25 | III | Straumann BLT | 4.1×8 |
|  | 12 | M | 32 | 25 | III | Straumann BLT | 4.1×10 |
|  | 13 | M | 56 | 16 | III | Straumann BLT | 4.8×8 |
|  | 13 | M | 56 | 17 | III | Straumann BLT | 4.8×8 |
|  | 14 | F | 36 | 22 | III | Straumann BLT | 3.3×12 |
|  | 15 | F | 47 | 16 | III | Straumann BLT | 4.8×10 |
|  | 16 | M | 77 | 16 | III | Straumann BLT | 4.8×10 |
|  | 16 | M | 77 | 17 | III | Straumann BLT | 4.8×10 |
|  | 17 | M | 56 | 37 | III | Astra EV | 4.8×8 |
|  | 18 | F | 32 | 13 | III | Astra EV | 3.6×11 |
|  | 19 | M | 60 | 17 | IV | Astra EV | 5.4×9 |
|  | 19 | M | 60 | 27 | III | Astra EV | 4.8×9 |
|  | 20 | F | 57 | 26 | III | Straumann BLT | 4.8×10 |
|  | 21 | F | 44 | 21 | III | Straumann BLT | 3.3×12 |
|  | 21 | F | 44 | 22 | III | Straumann BLT | 3.3×12 |
|  | 21 | F | 44 | 24 | III | Straumann BLT | 3.3×12 |
|  | 22 | F | 60 | 35 | III | Astra EV | 4.2×11 |
|  | 22 | F | 60 | 36 | III | Astra EV | 4.8×11 |
|  | 22 | F | 60 | 45 | III | Astra EV | 3.6×11 |
|  | 22 | F | 60 | 46 | III | Astra EV | 4.8×9 |
|  | 22 | F | 60 | 47 | III | Astra EV | 4.8×8 |
|  | 23 | M | 55 | 24 | III | Straumann BLT | 4.1×10 |
|  | 23 | M | 55 | 26 | III | Straumann BLT | 4.8×10 |
|  | 24 | F | 72 | 46 | III | Straumann BLT | 4.8×10 |
|  | 25 | M | 47 | 22 | III | Straumann BLT | 3.3×10 |
|  | 26 | F | 75 | 14 | III | Straumann BLT | 4.1×10 |
|  | 26 | F | 75 | 16 | III | Straumann BLT | 4.8×10 |
|  | 26 | F | 75 | 24 | III | Straumann BLT | 4.1×12 |
|  | 26 | F | 75 | 36 | III | Straumann BLT | 4.8×10 |
|  | 27 | F | 28 | 26 | III | Straumann BLT | 4.8×10 |
|  | 28 | F | 42 | 15 | III | Straumann BLT | 3.3×10 |
|  | 29 | F | 30 | 36 | III | Straumann BLT | 4.8×10 |
|  | 30 | F | 56 | 11 | III | Straumann BLT | 3.3×12 |
|  | 30 | F | 56 | 21 | III | Straumann BLT | 3.3×12 |
|  | 31 | M | 42 | 42 | III | Straumann BLT | 3.3×12 |
|  | 32 | M | 48 | 26 | III | Straumann BLT | 4.8×10 |
|  | 32 | M | 48 | 27 | III | Straumann BLT | 4.8×10 |
|  | 33 | M | 61 | 12 | III | Straumann BLT | 3.3×12 |
|  | 33 | M | 61 | 14 | III | Straumann BLT | 4.1×10 |
|  | 33 | M | 61 | 16 | III | Straumann BLT | 4.8×8 |
|  | 33 | M | 61 | 22 | III | Straumann BLT | 3.3×12 |
|  | 33 | M | 61 | 24 | III | Straumann BLT | 4.1×10 |
|  | 33 | M | 61 | 26 | III | Straumann BLT | 4.8×10 |
|  | 33 | M | 61 | 27 | III | Straumann BLT | 4.8×10 |
|  | 34 | F | 30 | 26 | III | Straumann BLT | 4.8×10 |
|  | 35 | M | 45 | 36 | III | Straumann BLT | 4.8×10 |
|  | 35 | M | 45 | 37 | III | Straumann BLT | 4.8×10 |
|  | 36 | F | 51 | 12 | III | Straumann BLT | 3.3×10 |
|  | 36 | F | 51 | 21 | III | Straumann BLT | 3.3×10 |
| I~II  (N=104; 60.1%) | 37 | M | 55 | 11 | II | Straumann BLT | 4.1×10 |
|  | 38 | F | 41 | 23 | II | Straumann BLT | 3.3×12 |
|  | 38 | F | 41 | 32 | II | Straumann BLT | 3.3×12 |
|  | 39 | F | 47 | 35 | II | Straumann BLT | 4.1×10 |
|  | 39 | F | 47 | 35 | II | Straumann BLT | 4.1×10 |
|  | 40 | F | 36 | 36 | II | Straumann BLT | 4.1×10 |
|  | 40 | F | 36 | 37 | II | Straumann BLT | 4.8×10 |
|  | 40 | F | 36 | 42 | II | Straumann BLT | 3.3×12 |
|  | 41 | F | 60 | 44 | II | Straumann BLT | 4.1×10 |
|  | 42 | F | 27 | 46 | II | Straumann BLT | 4.8×10 |
|  | 43 | M | 63 | 46 | II | Straumann BLT | 4.8×8 |
|  | 44 | M | 61 | 47 | II | Straumann BLT | 4.1×8 |
|  | 44 | M | 61 | 47 | II | Straumann BLT | 4.8×8 |
|  | 45 | F | 31 | 32 | II | Straumann BLT | 3.3×12 |
|  | 46 | F | 63 | 34 | II | Straumann BLT | 4.1×10 |
|  | 46 | F | 63 | 35 | II | Straumann BLT | 4.1×10 |
|  | 46 | F | 63 | 37 | II | Straumann BLT | 4.8×8 |
|  | 46 | F | 63 | 45 | II | Straumann BLT | 4.1×10 |
|  | 46 | F | 63 | 46 | II | Straumann BLT | 4.1×10 |
|  | 46 | F | 63 | 47 | II | Straumann BLT | 4.8×8 |
|  | 13 | M | 56 | 43 | II | Straumann BLT | 3.3×12 |
|  | 13 | M | 56 | 12 | II | Straumann BLT | 3.3×12 |
|  | 13 | M | 56 | 22 | II | Straumann BLT | 3.3×12 |
|  | 13 | M | 56 | 24 | II | Straumann BLT | 4.1×10 |
|  | 13 | M | 56 | 26 | II | Straumann BLT | 4.8×10 |
|  | 13 | M | 56 | 27 | II | Straumann BLT | 4.8×10 |
|  | 13 | M | 56 | 14 | II | Straumann BLT | 4.1×10 |
|  | 22 | F | 60 | 34 | II | Astra EV | 3.0×13 |
|  | 47 | F | 54 | 47 | II | Straumann BLT | 4.8×10 |
|  | 48 | F | 40 | 36 | II | Straumann BLT | 4.8×10 |
|  | 49 | F | 35 | 12 | II | Straumann BLT | 3.3×12 |
|  | 49 | F | 35 | 22 | II | Straumann BLT | 3.3×12 |
|  | 50 | F | 34 | 21 | II | Straumann BLT | 3.3×12 |
|  | 51 | F | 28 | 46 | II | Straumann BLT | 4.8×10 |
|  | 52 | F | 20 | 26 | II | Straumann BLT | 4.8×10 |
|  | 53 | F | 52 | 46 | II | Straumann BLT | 4.8×10 |
|  | 54 | F | 51 | 23 | II | Straumann BLT | 4.1×10 |
|  | 55 | M | 61 | 26 | II | Straumann BLT | 4.8×10 |
|  | 55 | M | 61 | 42 | II | Straumann BLT | 3.3×12 |
|  | 56 | F | 32 | 36 | II | Straumann BLT | 4.8×10 |
|  | 56 | F | 32 | 46 | II | Straumann BLT | 4.8×10 |
|  | 57 | M | 39 | 17 | II | Straumann BLT | 4.8×10 |
|  | 57 | M | 39 | 32 | II | Straumann BLT | 3.3×12 |
|  | 57 | M | 39 | 42 | II | Straumann BLT | 3.3×12 |
|  | 58 | M | 45 | 36 | II | Astra EV | 4.8×9 |
|  | 58 | M | 45 | 37 | II | Astra EV | 4.8×8 |
|  | 59 | F | 32 | 36 | II | Straumann BLT | 4.8×10 |
|  | 60 | M | 42 | 25 | II | Straumann BLT | 3.3×10 |
|  | 60 | M | 42 | 36 | II | Straumann BLT | 4.8×10 |
|  | 61 | M | 43 | 46 | II | Straumann BLT | 4.8×8 |
|  | 62 | M | 38 | 43 | II | Straumann BLT | 4.1×10 |
|  | 62 | M | 38 | 44 | II | Straumann BLT | 4.1×10 |
|  | 62 | M | 38 | 45 | II | Straumann BLT | 4.8×10 |
|  | 63 | M | 26 | 46 | II | Astra EV | 4.8×11 |
|  | 64 | F | 53 | 16 | II | Straumann BLT | 4.8×10 |
|  | 64 | F | 53 | 17 | II | Straumann BLT | 4.8×10 |
|  | 65 | F | 65 | 45 | II | Straumann BLT | 4.1×10 |
|  | 65 | F | 65 | 47 | II | Straumann BLT | 4.8×10 |
|  | 66 | F | 28 | 13 | II | Straumann BLT | 3.3×12 |
|  | 66 | F | 28 | 23 | II | Straumann BLT | 3.3×12 |
|  | 67 | M | 59 | 46 | II | Straumann BLT | 4.8×10 |
|  | 67 | M | 59 | 47 | II | Straumann BLT | 4.8×10 |
|  | 68 | F | 38 | 46 | II | Straumann BLT | 4.8×10 |
|  | 68 | F | 38 | 47 | II | Straumann BLT | 4.8×8 |
|  | 25 | M | 47 | 36 | II | Straumann BLT | 4.8×10 |
|  | 25 | M | 47 | 37 | II | Straumann BLT | 4.8×10 |
|  | 69 | M | 60 | 32 | II | Straumann BLT | 3.3×10 |
|  | 69 | M | 60 | 42 | II | Straumann BLT | 3.3×12 |
|  | 70 | M | 39 | 36 | II | Straumann BLT | 4.8×10 |
|  | 71 | F | 48 | 47 | II | Straumann BLT | 4.8×10 |
|  | 72 | F | 31 | 36 | II | Straumann BLT | 4.8×10 |
|  | 72 | F | 31 | 46 | II | Straumann BLT | 4.8×10 |
|  | 73 | F | 30 | 12 | II | Straumann BLT | 3.3×12 |
|  | 74 | M | 61 | 34 | II | Straumann BLT | 4.1×10 |
|  | 74 | M | 61 | 36 | II | Straumann BLT | 4.8×10 |
|  | 74 | M | 61 | 44 | II | Straumann BLT | 4.1×10 |
|  | 74 | M | 61 | 46 | II | Straumann BLT | 4.8×10 |
|  | 75 | M | 55 | 36 | II | Straumann BLT | 4.8×10 |
|  | 75 | M | 55 | 47 | II | Straumann BLT | 4.8×10 |
|  | 33 | M | 61 | 32 | II | Straumann BLT | 3.3×12 |
|  | 33 | M | 61 | 34 | II | Straumann BLT | 4.1×10 |
|  | 33 | M | 61 | 36 | II | Straumann BLT | 4.8×8 |
|  | 33 | M | 61 | 42 | II | Straumann BLT | 3.3×12 |
|  | 33 | M | 61 | 44 | II | Straumann BLT | 4.1×10 |
|  | 33 | M | 61 | 46 | II | Straumann BLT | 4.8×10 |
|  | 76 | F | 42 | 42 | I | Straumann BLT | 3.3×12 |
|  | 76 | F | 42 | 42 | I | Straumann BLT | 3.3×12 |
|  | 77 | M | 70 | 46 | I | BEGO SCX | 4.5×10 |
|  | 77 | M | 70 | 47 | I | BEGO SCX | 4.5×10 |
|  | 13 | M | 56 | 44 | I | Straumann BLT | 4.1×10 |
|  | 13 | M | 56 | 46 | I | Straumann BLT | 4.8×10 |
|  | 13 | M | 56 | 47 | I | Straumann BLT | 4.8×10 |
|  | 13 | M | 56 | 35 | I | Straumann BLT | 4.1×10 |
|  | 13 | M | 56 | 36 | I | Straumann BLT | 4.8×10 |
|  | 13 | M | 56 | 37 | I | Straumann BLT | 4.8×8 |
|  | 78 | F | 21 | 21 | I | Straumann BLT | 3.3×10 |
|  | 79 | F | 32 | 14 | I | Straumann BLT | 3.3×12 |
|  | 79 | F | 32 | 15 | I | Straumann BLT | 4.1×12 |
|  | 80 | M | 47 | 37 | I | Straumann BLT | 4.8×10 |
|  | 81 | M | 61 | 46 | I | Astra EV | 4.8×9 |
|  | 81 | M | 61 | 47 | I | Astra EV | 4.8×9 |
|  | 82 | M | 61 | 32 | I | Straumann BLT | 3.3×12 |
|  | 82 | M | 61 | 42 | I | Straumann BLT | 3.3×12 |
|  | 83 | F | 67 | 26 | I | Straumann BLT | 4.8×10 |

Abbreviations: F, female; M, male; mm, millimeter.

**Supplementary Table 2. Participant demographic and surgical information** **divided into four groups by implant site**

| Implant sites Number of samples,N; Proportion(%) | Subject No. | Sex | Age  (Years old) | Implant location | Bone quality  type | Company | Implant Diameter×Length (mm) |
| --- | --- | --- | --- | --- | --- | --- | --- |
| UA  (N=38; 22%) | 2 | M | 23 | 11 | III | Straumann BLT | 3.3×10 |
|  | 4 | F | 40 | 11 | IV | Straumann BLT | 3.3×10 |
|  | 5 | F | 43 | 11 | IV | Straumann BLT | 3.3×12 |
|  | 6 | F | 65 | 11 | IV | Straumann BLT | 3.3×12 |
|  | 37 | M | 55 | 11 | II | Straumann BLT | 4.1×10 |
|  | 30 | F | 56 | 11 | III | Straumann BLT | 3.3×12 |
|  | 6 | F | 65 | 12 | IV | Straumann BLT | 3.3×10 |
|  | 13 | M | 56 | 12 | II | Straumann BLT | 3.3×12 |
|  | 49 | F | 35 | 12 | II | Straumann BLT | 3.3×12 |
|  | 73 | F | 30 | 12 | II | Straumann BLT | 3.3×12 |
|  | 33 | M | 61 | 12 | III | Straumann BLT | 3.3×12 |
|  | 36 | F | 51 | 12 | III | Straumann BLT | 3.3×10 |
|  | 18 | F | 32 | 13 | III | Astra EV | 3.6×11 |
|  | 66 | F | 28 | 13 | II | Straumann BLT | 3.3×12 |
|  | 6 | F | 65 | 14 | IV | Straumann BLT | 3.3×10 |
|  | 13 | M | 56 | 14 | II | Straumann BLT | 4.1×10 |
|  | 79 | F | 32 | 14 | I | Straumann BLT | 3.3×12 |
|  | 26 | F | 75 | 14 | III | Straumann BLT | 4.1×10 |
|  | 33 | M | 61 | 14 | III | Straumann BLT | 4.1×10 |
|  | 1 | F | 31 | 21 | III | Straumann BLT | 3.3×10 |
|  | 2 | M | 23 | 21 | III | Straumann BLT | 3.3×10 |
|  | 7 | F | 53 | 21 | IV | Straumann BLT | 3.3×12 |
|  | 10 | M | 60 | 21 | III | Straumann BLT | 3.3×12 |
|  | 21 | F | 44 | 21 | III | Straumann BLT | 3.3×12 |
|  | 78 | F | 21 | 21 | I | Straumann BLT | 3.3×10 |
|  | 50 | F | 34 | 21 | II | Straumann BLT | 3.3×12 |
|  | 30 | F | 56 | 21 | III | Straumann BLT | 3.3×12 |
|  | 36 | F | 51 | 21 | III | Straumann BLT | 3.3×10 |
|  | 13 | M | 56 | 22 | II | Straumann BLT | 3.3×12 |
|  | 14 | F | 36 | 22 | III | Straumann BLT | 3.3×12 |
|  | 21 | F | 44 | 22 | III | Straumann BLT | 3.3×12 |
|  | 49 | F | 35 | 22 | II | Straumann BLT | 3.3×12 |
|  | 25 | M | 47 | 22 | III | Straumann BLT | 3.3×10 |
|  | 33 | M | 61 | 22 | III | Straumann BLT | 3.3×12 |
|  | 8 | F | 54 | 23 | IV | Straumann BLT | 3.3×10 |
|  | 38 | F | 41 | 23 | II | Straumann BLT | 3.3×12 |
|  | 54 | F | 51 | 23 | II | Straumann BLT | 4.1×10 |
|  | 66 | F | 28 | 23 | II | Straumann BLT | 3.3×12 |
| UP  (N=44; 25.4%) | 79 | F | 32 | 15 | I | Straumann BLT | 4.1×12 |
|  | 28 | F | 42 | 15 | III | Straumann BLT | 3.3×10 |
|  | 3 | M | 47 | 16 | III | Straumann BLT | 4.8×10 |
|  | 6 | F | 65 | 16 | IV | Straumann BLT | 4.8×10 |
|  | 13 | M | 56 | 16 | III | Straumann BLT | 4.8×8 |
|  | 15 | F | 47 | 16 | III | Straumann BLT | 4.8×10 |
|  | 16 | M | 77 | 16 | III | Straumann BLT | 4.8×10 |
|  | 64 | F | 53 | 16 | II | Straumann BLT | 4.8×10 |
|  | 26 | F | 75 | 16 | III | Straumann BLT | 4.8×10 |
|  | 33 | M | 61 | 16 | III | Straumann BLT | 4.8×8 |
|  | 7 | F | 53 | 17 | IV | Straumann BLT | 4.8×10 |
|  | 7 | F | 53 | 17 | IV | Straumann BLT | 4.8×8 |
|  | 13 | M | 56 | 17 | III | Straumann BLT | 4.8×8 |
|  | 16 | M | 77 | 17 | III | Straumann BLT | 4.8×10 |
|  | 19 | M | 60 | 17 | IV | Astra EV | 5.4×9 |
|  | 57 | M | 39 | 17 | II | Straumann BLT | 4.8×10 |
|  | 64 | F | 53 | 17 | II | Straumann BLT | 4.8×10 |
|  | 8 | F | 54 | 24 | IV | Straumann BLT | 4.1×10 |
|  | 13 | M | 56 | 24 | II | Straumann BLT | 4.1×10 |
|  | 21 | F | 44 | 24 | III | Straumann BLT | 3.3×12 |
|  | 23 | M | 55 | 24 | III | Straumann BLT | 4.1×10 |
|  | 26 | F | 75 | 24 | III | Straumann BLT | 4.1×12 |
|  | 33 | M | 61 | 24 | III | Straumann BLT | 4.1×10 |
|  | 8 | F | 54 | 25 | IV | Straumann BLT | 4.1×10 |
|  | 11 | F | 24 | 25 | III | Straumann BLT | 4.1×8 |
|  | 12 | M | 32 | 25 | III | Straumann BLT | 4.1×10 |
|  | 60 | M | 42 | 25 | II | Straumann BLT | 3.3×10 |
|  | 9 | F | 43 | 26 | IV | Straumann BLT | 4.8×8 |
|  | 13 | M | 56 | 26 | II | Straumann BLT | 4.8×10 |
|  | 20 | F | 57 | 26 | III | Straumann BLT | 4.8×10 |
|  | 52 | F | 20 | 26 | II | Straumann BLT | 4.8×10 |
|  | 55 | M | 61 | 26 | II | Straumann BLT | 4.8×10 |
|  | 23 | M | 55 | 26 | III | Straumann BLT | 4.8×10 |
|  | 27 | F | 28 | 26 | III | Straumann BLT | 4.8×10 |
|  | 32 | M | 48 | 26 | III | Straumann BLT | 4.8×10 |
|  | 33 | M | 61 | 26 | III | Straumann BLT | 4.8×10 |
|  | 83 | F | 67 | 26 | I | Straumann BLT | 4.8×10 |
|  | 34 | F | 30 | 26 | III | Straumann BLT | 4.8×10 |
|  | 9 | F | 43 | 27 | IV | Straumann BLT | 4.8×10 |
|  | 9 | F | 43 | 27 | IV | Straumann BLT | 4.8×8 |
|  | 13 | M | 56 | 27 | II | Straumann BLT | 4.8×10 |
|  | 19 | M | 60 | 27 | III | Astra EV | 4.8×9 |
|  | 32 | M | 48 | 27 | III | Straumann BLT | 4.8×10 |
|  | 33 | M | 61 | 27 | III | Straumann BLT | 4.8×10 |
| LA  (N=17; 9.8%) | 38 | F | 41 | 32 | II | Straumann BLT | 3.3×12 |
|  | 45 | F | 31 | 32 | II | Straumann BLT | 3.3×12 |
|  | 57 | M | 39 | 32 | II | Straumann BLT | 3.3×12 |
|  | 69 | M | 60 | 32 | II | Straumann BLT | 3.3×10 |
|  | 82 | M | 61 | 32 | I | Straumann BLT | 3.3×12 |
|  | 33 | M | 61 | 32 | II | Straumann BLT | 3.3×12 |
|  | 40 | F | 36 | 42 | II | Straumann BLT | 3.3×12 |
|  | 76 | F | 42 | 42 | I | Straumann BLT | 3.3×12 |
|  | 76 | F | 42 | 42 | I | Straumann BLT | 3.3×12 |
|  | 55 | M | 61 | 42 | II | Straumann BLT | 3.3×12 |
|  | 57 | M | 39 | 42 | II | Straumann BLT | 3.3×12 |
|  | 69 | M | 60 | 42 | II | Straumann BLT | 3.3×12 |
|  | 82 | M | 61 | 42 | I | Straumann BLT | 3.3×12 |
|  | 31 | M | 42 | 42 | III | Straumann BLT | 3.3×12 |
|  | 33 | M | 61 | 42 | II | Straumann BLT | 3.3×12 |
|  | 13 | M | 56 | 43 | II | Straumann BLT | 3.3×12 |
|  | 62 | M | 38 | 43 | II | Straumann BLT | 4.1×10 |
| LP  (N=74; 42.8%) | 41 | F | 60 | 44 | II | Straumann BLT | 4.1×10 |
|  | 13 | M | 56 | 44 | I | Straumann BLT | 4.1×10 |
|  | 62 | M | 38 | 44 | II | Straumann BLT | 4.1×10 |
|  | 74 | M | 61 | 44 | II | Straumann BLT | 4.1×10 |
|  | 33 | M | 61 | 44 | II | Straumann BLT | 4.1×10 |
|  | 46 | F | 63 | 45 | II | Straumann BLT | 4.1×10 |
|  | 22 | F | 60 | 45 | III | Astra EV | 3.6×11 |
|  | 62 | M | 38 | 45 | II | Straumann BLT | 4.8×10 |
|  | 65 | F | 65 | 45 | II | Straumann BLT | 4.1×10 |
|  | 42 | F | 27 | 46 | II | Straumann BLT | 4.8×10 |
|  | 43 | M | 63 | 46 | II | Straumann BLT | 4.8×8 |
|  | 77 | M | 70 | 46 | I | BEGO SCX | 4.5×10 |
|  | 46 | F | 63 | 46 | II | Straumann BLT | 4.1×10 |
|  | 13 | M | 56 | 46 | I | Straumann BLT | 4.8×10 |
|  | 22 | F | 60 | 46 | III | Astra EV | 4.8×9 |
|  | 51 | F | 28 | 46 | II | Straumann BLT | 4.8×10 |
|  | 53 | F | 52 | 46 | II | Straumann BLT | 4.8×10 |
|  | 56 | F | 32 | 46 | II | Straumann BLT | 4.8×10 |
|  | 61 | M | 43 | 46 | II | Straumann BLT | 4.8×8 |
|  | 63 | M | 26 | 46 | II | Astra EV | 4.8×11 |
|  | 67 | M | 59 | 46 | II | Straumann BLT | 4.8×10 |
|  | 24 | F | 72 | 46 | III | Straumann BLT | 4.8×10 |
|  | 68 | F | 38 | 46 | II | Straumann BLT | 4.8×10 |
|  | 81 | M | 61 | 46 | I | Astra EV | 4.8×9 |
|  | 72 | F | 31 | 46 | II | Straumann BLT | 4.8×10 |
|  | 74 | M | 61 | 46 | II | Straumann BLT | 4.8×10 |
|  | 33 | M | 61 | 46 | II | Straumann BLT | 4.8×10 |
|  | 44 | M | 61 | 47 | II | Straumann BLT | 4.1×8 |
|  | 44 | M | 61 | 47 | II | Straumann BLT | 4.8×8 |
|  | 77 | M | 70 | 47 | I | BEGO SCX | 4.5×10 |
|  | 46 | F | 63 | 47 | II | Straumann BLT | 4.8×8 |
|  | 13 | M | 56 | 47 | I | Straumann BLT | 4.8×10 |
|  | 22 | F | 60 | 47 | III | Astra EV | 4.8×8 |
|  | 47 | F | 54 | 47 | II | Straumann BLT | 4.8×10 |
|  | 65 | F | 65 | 47 | II | Straumann BLT | 4.8×10 |
|  | 67 | M | 59 | 47 | II | Straumann BLT | 4.8×10 |
|  | 68 | F | 38 | 47 | II | Straumann BLT | 4.8×8 |
|  | 81 | M | 61 | 47 | I | Astra EV | 4.8×9 |
|  | 71 | F | 48 | 47 | II | Straumann BLT | 4.8×10 |
|  | 75 | M | 55 | 47 | II | Straumann BLT | 4.8×10 |
|  | 46 | F | 63 | 34 | II | Straumann BLT | 4.1×10 |
|  | 22 | F | 60 | 34 | II | Astra EV | 3.0×13 |
|  | 74 | M | 61 | 34 | II | Straumann BLT | 4.1×10 |
|  | 33 | M | 61 | 34 | II | Straumann BLT | 4.1×10 |
|  | 39 | F | 47 | 35 | II | Straumann BLT | 4.1×10 |
|  | 39 | F | 47 | 35 | II | Straumann BLT | 4.1×10 |
|  | 46 | F | 63 | 35 | II | Straumann BLT | 4.1×10 |
|  | 13 | M | 56 | 35 | I | Straumann BLT | 4.1×10 |
|  | 22 | F | 60 | 35 | III | Astra EV | 4.2×11 |
|  | 40 | F | 36 | 36 | II | Straumann BLT | 4.1×10 |
|  | 13 | M | 56 | 36 | I | Straumann BLT | 4.8×10 |
|  | 22 | F | 60 | 36 | III | Astra EV | 4.8×11 |
|  | 48 | F | 40 | 36 | II | Straumann BLT | 4.8×10 |
|  | 56 | F | 32 | 36 | II | Straumann BLT | 4.8×10 |
|  | 58 | M | 45 | 36 | II | Astra EV | 4.8×9 |
|  | 59 | F | 32 | 36 | II | Straumann BLT | 4.8×10 |
|  | 60 | M | 42 | 36 | II | Straumann BLT | 4.8×10 |
|  | 25 | M | 47 | 36 | II | Straumann BLT | 4.8×10 |
|  | 26 | F | 75 | 36 | III | Straumann BLT | 4.8×10 |
|  | 70 | M | 39 | 36 | II | Straumann BLT | 4.8×10 |
|  | 29 | F | 30 | 36 | III | Straumann BLT | 4.8×10 |
|  | 72 | F | 31 | 36 | II | Straumann BLT | 4.8×10 |
|  | 74 | M | 61 | 36 | II | Straumann BLT | 4.8×10 |
|  | 75 | M | 55 | 36 | II | Straumann BLT | 4.8×10 |
|  | 33 | M | 61 | 36 | II | Straumann BLT | 4.8×8 |
|  | 35 | M | 45 | 36 | III | Straumann BLT | 4.8×10 |
|  | 40 | F | 36 | 37 | II | Straumann BLT | 4.8×10 |
|  | 46 | F | 63 | 37 | II | Straumann BLT | 4.8×8 |
|  | 13 | M | 56 | 37 | I | Straumann BLT | 4.8×8 |
|  | 17 | M | 56 | 37 | III | Astra EV | 4.8×8 |
|  | 80 | M | 47 | 37 | I | Straumann BLT | 4.8×10 |
|  | 58 | M | 45 | 37 | II | Astra EV | 4.8×8 |
|  | 25 | M | 47 | 37 | II | Straumann BLT | 4.8×10 |
|  | 35 | M | 45 | 37 | III | Straumann BLT | 4.8×10 |

Abbreviations: F, female; M, male; mm, millimeter.
