# Supplementary material for: Impact of Implant Site and Bone Quality on the Accuracy of Robot‐Assisted Implant Placement: A Retrospective Study
Source: Int J Dent. 2026 Feb 17;2026:3947015. doi: 10.1155/ijod/3947015 (PMC12913689; doi:10.1155/ijod/3947015)
Supplement: Supplementary file 2 — Supporting Information 2 Table S2: Participant demographic and surgical information divided into four groups by implant site. [file IJOD-2026-3947015-s001.docx]

Table 1 The analysis of homogeneity of variance in UP, LP, UA and LA

|  |  | UP **(N=44)** | LP **(N=74)** | UA **(N=38)** | LA **(N=17)** | **P value** |
| --- | --- | --- | --- | --- | --- | --- |
| LCD | Median | 0.310 | 0.325 | 0.350 | 0.360 | 0.3977^b^ |
|  | Q1,Q3 | 0.200, 0.420 | 0.250, 0.420 | 0.200, 0.390 | 0.260, 0.530 |  |
|  | Min,Max | 0.04, 0.90 | 0.07, 0.95 | 0.05, 0.91 | 0.08, 0.77 |  |
| VCD | Median | 0.510 | 0.225 | 0.750 | 0.200 | <0.0001^a^ |
|  | Q1,Q3 | 0.140, 0.849 | -0.090, 0.440 | 0.590, 1.180 | -0.020, 0.740 |  |
|  | Min,Max | -0.65, 1.64 | -0.71, 1.61 | 0.11, 1.93 | -0.25, 1.09 |  |
| GCD | Median | 0.660 | 0.510 | 0.800 | 0.540 | <0.0001^b^ |
|  | Q1,Q3 | 0.440, 0.900 | 0.340, 0.740 | 0.640, 1.210 | 0.320, 0.920 |  |
|  | Min,Max | 0.28, 1.68 | 0.13, 1.66 | 0.40, 2.03 | 0.14, 1.30 |  |
| LAD | Median | 0.350 | 0.347 | 0.360 | 0.450 | 0.0326^b^ |
|  | Q1,Q3 | 0.270, 0.430 | 0.260, 0.470 | 0.230, 0.420 | 0.360, 0.550 |  |
|  | Min,Max | 0.07, 1.07 | 0.04, 1.22 | 0.08, 0.69 | 0.21, 0.94 |  |
| VAD | Median | 0.490 | 0.220 | 0.740 | 0.200 | <0.0001^a^ |
|  | Q1,Q3 | 0.130, 0.830 | -0.070, 0.430 | 0.590, 1.180 | -0.020, 0.740 |  |
|  | Min,Max | -0.66, 1.64 | -0.72, 1.61 | 0.11, 1.92 | -0.25, 1.08 |  |
| GAD | Median | 0.670 | 0.515 | 0.850 | 0.630 | 0.0003^b^ |
|  | Q1,Q3 | 0.488, 0.935 | 0.400, 0.730 | 0.660, 1.230 | 0.500, 0.970 |  |
|  | Min,Max | 0.19, 1.68 | 0.08, 1.66 | 0.19, 1.97 | 0.37, 1.39 |  |
| AD | Median | 1.250 | 1.150 | 1.240 | 0.950 | 0.5068^b^ |
|  | Q1,Q3 | 0.800, 1.750 | 0.620, 1.560 | 0.970, 1.520 | 0.630, 1.560 |  |
|  | Min,Max | 0.19, 3.90 | 0.05, 6.47 | 0.11, 2.10 | 0.18, 188.00 |  |
| Note: Data were analyzed using analysis of variance or Kruskal-Wallis test. "a" represents analysis of variance and "b" represents Kruskal-Wallis test. | | | | | | |

**Table 2** The analysis of homogeneity of variance in I-II and III-IV

|  |  | **I-II (N=104)** | **III-IV (N=69)** | **P value** |
| --- | --- | --- | --- | --- |
| LCD | Median | 0.320 | 0.340 | 0.9748^b^ |
|  | Q1,Q3 | 0.220, 0.420 | 0.240, 0.430 |  |
|  | Min,Max | 0.05, 0.95 | 0.04, 0.91 |  |
| VCD | Median | 0.230 | 0.600 | <0.0001^a^ |
|  | Q1,Q3 | -0.050, 0.620 | 0.390, 0.900 |  |
|  | Min,Max | -0.71, 1.27 | -0.54, 1.93 |  |
| GCD | Median | 0.530 | 0.700 | 0.0003^b^ |
|  | Q1,Q3 | 0.390, 0.800 | 0.540, 1.070 |  |
|  | Min,Max | 0.13, 1.33 | 0.31, 2.03 |  |
| LAD | Median | 0.370 | 0.360 | 0.3653^b^ |
|  | Q1,Q3 | 0.260, 0.490 | 0.280, 0.428 |  |
|  | Min,Max | 0.04, 1.22 | 0.10, 1.07 |  |
| VAD | Median | 0.220 | 0.600 | <0.0001^a^ |
|  | Q1,Q3 | -0.050, 0.620 | 0.390, 0.890 |  |
|  | Min,Max | -0.72, 1.26 | -0.54, 1.92 |  |
| GAD | Median | 0.603 | 0.720 | 0.0037^b^ |
|  | Q1,Q3 | 0.450, 0.830 | 0.550, 1.140 |  |
|  | Min,Max | 0.08, 1.39 | 0.19, 1.97 |  |
| AD | Median | 1.220 | 1.230 | 0.3242^b^ |
|  | Q1,Q3 | 0.700, 1.560 | 0.800, 1.680 |  |
|  | Min,Max | 0.05, 188.00 | 0.11, 3.92 |  |
| Note: Data were analyzed using analysis of variance or Kruskal-Wallis test. "a" represents analysis of variance and "b" represents Kruskal-Wallis test. | | | | |
